# Insights into diet and niche separation in the context of prey availability between two sympatric whiprays in a tropical intertidal estuary

**DOI:** 10.1111/jfb.70596

**Published:** 2026-08-02

**Authors:** Jaelen Myers, Marcus Sheaves, Adam Barnett

**Affiliations:** ^1^ James Cook University Townsville Queensland Australia; ^2^ Centre for Tropical Water and Aquatic Ecosystem Research (TropWATER) James Cook University Cairns Queensland Australia; ^3^ Biopixel Oceans Foundation Cairns Queensland Australia

**Keywords:** Australia, *Himantura australis*, *Maculabatis toshi*, resource partitioning, stable isotope, trophic ecology

## Abstract

Increasing evidence supports associations between various elasmobranch species and estuarine habitats, particularly during early life stages. However, with substantial knowledge gaps surrounding spatial habitat use and trophic ecology of estuary‐associated species in Australia, we cannot reliably assess the functional importance of these areas or identify relevant drivers of resource partitioning in estuarine food webs. Here, we compared the diets and trophic niches of two sympatric dasyatid species, the Australian whipray *Himantura australis* and brown whipray *Maculabatis toshi,* in a tropical estuary in North Queensland. Stomach contents were collected from juvenile specimens in 2022–2025 using gastric lavage, muscle tissue samples were analysed for stable isotopes (δ^13^C, δ^15^N) and invertebrate communities were surveyed to contextualise prey availability in the intertidal zone. Dietary composition differed significantly between species, with *H. australis* showing a stronger reliance on burrowing crustaceans (e.g., Callianassidae and Mictyridae), while *M. toshi* consumed more epibenthic crustaceans (e.g., Ogyrididae, Penaeidae, Portunidae). *Himantura australis* occupied a larger isotopic niche space than *M. toshi*, and there was no trophic overlap of larger size classes of *H. australis* (>50 cm disc width) with *M. toshi* or with smaller conspecifics (<50 cm disc width). Prey availability was spatially variable and indicated that while both species target locally abundant crustacean taxa, resources may be partitioned across different sediment layers. These findings highlight the importance of estuaries as feeding grounds for juvenile rays and suggest that morphological and behavioural adaptations may drive dietary variability between similar species.

## 1 INTRODUCTION

Estuaries are highly dynamic and productive ecosystems shaped by the convergence of freshwater and marine inputs (Kennish, 2002). They are also among the most threatened coastal ecosystems globally, facing unprecedented pressures from expanding human populations, coastal development, pollution and climate change (Leal Filho et al., 2022; Lotze et al., 2006; McLusky & Elliott, 2004). These transitional zones support diverse assemblages of marine life, including transient predators, that exploit intertidal zones and mangrove ecotones during tidal inundations (Krumme, 2009; Reis‐Filho et al., 2019; Sheaves et al., 2024). Many estuaries serve as essential fish habitats by offering abundant foraging opportunities and refuge from predation (Nagelkerken et al., 2015; Whitfield, 2017). With resource availability and accessibility being fundamental determinants of estuarine habitat quality (Davis et al., 2022; Saulnier et al., 2020), it is vital to understand how species' feeding ecologies shape their habitat use and trophic interactions.

Sharks and rays fulfil important functional roles in marine and estuarine ecosystems (Barnett et al., 2017; Flowers et al., 2021; Heithaus et al., 2010; Heupel et al., 2014). These roles are bidirectional for mesopredators, in which their presence influences the behaviours, distributions and abundances of their prey while simultaneously serving as prey for higher trophic levels (Bond et al., 2019; Heithaus & Vaudo, 2012). The roles of benthic ray species are particularly relevant in nearshore tidal areas, where excavation‐style foraging behaviours contribute to bioturbation and ecosystem engineering processes (Flowers et al., 2021; Grew et al., 2024; Nauta et al., 2024). While increasing evidence supports the reliance of various ray species on estuarine environments, substantial knowledge gaps persist regarding species‐specific habitat use and feeding strategies (Constance et al., 2024; Grant et al., 2019).

Stingrays (Dasyatidae) are often considered generalist predators that consume a variety of invertebrates including crabs, shrimps, annelids and molluscs (Flowers et al., 2021). However, differences in diets which are common among species and intraspecific populations (by age, sex or across habitats) can create dietary specialisation towards specific taxa (Collins et al., 2007; Munroe et al., 2014). Studies often show interspecific dietary separation and/or fine‐scale spatial partitioning among species that share the same habitats, which may facilitate coexistence by reducing competition for limited resources (Elston et al., 2020; Hayata et al., 2021; Mulas et al., 2019; Pardo et al., 2015). Spatial resource partitioning can occur horizontally when species select different microhabitats for feeding (Crook et al., 2022; Myers et al., 2026) or vertically by targeting prey across different sediment depths (Myers, Sheaves, Abrantes, et al., 2025; Platell et al., 1998). Morphological adaptations, such as dentition structures and rostra shape, also facilitate the handling of certain prey types and mechanical excavation capabilities (Kolmann et al., 2023; Pardo et al., 2015). Within populations, ontogenetic dietary shifts are expected as individuals grow and exploit new habitats and resource pools (Barbini & Lucifora, 2012; Elston et al., 2020; Yogi et al., 2023). Moreover, with dietary variability further subject to spatiotemporal prey availability (particularly for generalist predators), it becomes necessary to interpret dietary patterns within the appropriate habitat context (Every et al., 2019; Munroe et al., 2015).

Continued investigations of diets among sympatric species are needed to identify specific drivers of resource partitioning and to understand underlying relationships between prey consumption, dietary specialisation and resource availability (Munroe et al., 2014). Two widely distributed and morphologically similar dasyatid whipray species, the Australian whipray *Himantura australis* Last, White & Naylor 2016 and brown whipray *Maculabatis toshi* (Whitley 1939), have overlapping distributions throughout the Australian tropics (Last, Naylor, et al., 2016). Both species have similar habitat preferences, with juveniles generally inhabiting shallow marine or estuarine areas (Rigby & Derrick, 2021; Rigby & Pierce, 2016). Limited evidence from other habitat types supports that while each species consistently ingests decapod crustacean prey, their dietary and trophic niches may differ (Myers, Sheaves, Abrantes, et al., 2025; Vaudo & Heithaus, 2011; Wagiyo et al., 2023). However, relatively little is known about the feeding ecology of either species during early juvenile stages or from an estuary context. In this study, we used stomach content analysis and stable isotope analysis to compare the diets and trophic niche overlap between *H. australis* and *M. toshi* i a tropical estuary inlet in northeastern Australia. Benthic invertebrates were also sampled to assess the spatial distributions of prey resources across the intertidal zone and to contextualise prey selection of each species.

## 2 MATERIALS AND METHODS

### 2.1 Study site

Blacksoil Creek (19.299407° S, 147.042662° E) is an estuary inlet located in Cape Cleveland, North Queensland, Australia, adjacent to Bowling Green National Park (Figure 1a). The site experiences a semi‐diurnal tidal regime with a maximum tidal range of approximately 4 m above Lowest Astronomical Tide. The creek channel is surrounded by saltpans, which contain minimal urban development or farmland in its immediate catchment (Mattone & Sheaves, 2017). The study site includes 0.5 km^2^ near the estuary opening, with habitat largely comprising intertidal sand flats surrounded by deeper subtidal channels along the mangrove edges, primarily of *Avicennia marina* and *Rhizophora stylosa*. The homogenous sand flats are densely covered with ray feeding pits, which are readily visible at low tide. During standard low tides (<1.5 m), most of the intertidal flats are exposed, but these become regularly submerged at tidal heights >2.5 m, allowing rays to access them for feeding (Figure 1b). The site experiences some delay in flooding due to the tidal flow being partially blocked by sandbars outside the estuary opening, which causes water depth in the creek to rise quickly, particularly during spring tidal phases.

**FIGURE 1:**
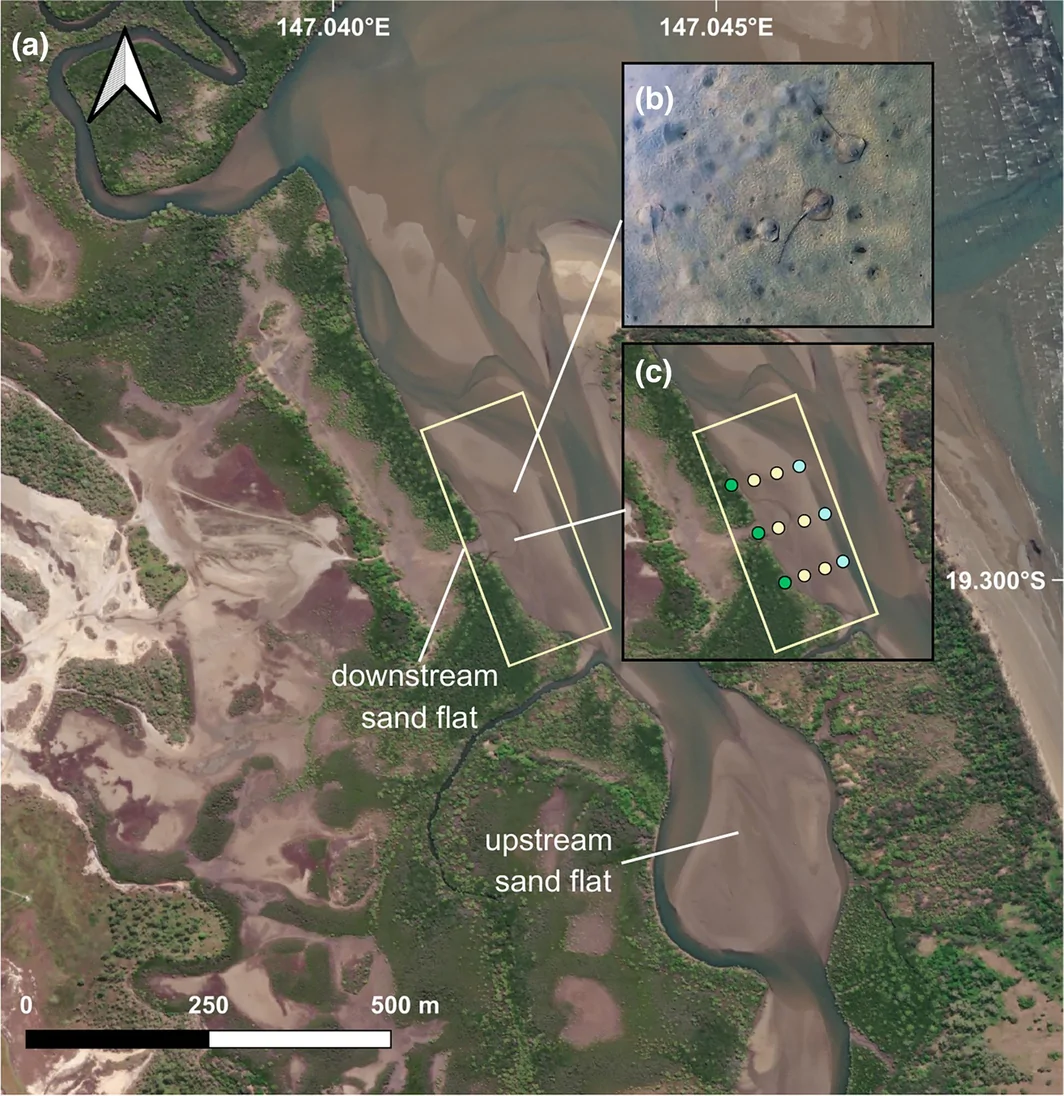
Satellite overview of the Blacksoil Creek estuary at low tide. (a) The inset in panel b shows a cluster of *Himantura australis* feeding on the submerged intertidal flat area (b). Within the yellow box (c), points mark the 12 sites where benthic fauna sampling was conducted on the downstream sand flat, with colours indicating the habitat stratum: Green = mangrove edge, yellow = open sand flat, blue = subtidal edge. Satellite map: ESRI World Imagery.

### 2.2 Ethics statement

All handling and collections from ray specimens were carried out under approval by James Cook University [Animal Ethics Approval #A2838].

### 2.3 Gastric lavage and tissue collection

Rays were captured over 17 sampling trips from February 2022 to June 2025 primarily during low, early rising and late ebb daytime tides when water levels were low enough for the intertidal areas to be partially or wholly exposed. Rays were spotted in shallow water (<1 m) and captured by small teams wading into the water and encircling them with a beach seine net. Each ray was then transferred from the seine net to handheld dip nets. Disc widths of *H. australis* in this study ranged from 30 to 80 cm DW (mean 52.7 ± 3.2 cm), representing young‐of‐year to later stage juveniles (~4–5 years) based on known age‐growth relationships (Last, White, & Naylor, 2016; O'Shea, Braccini, et al., 2013). For *M. toshi*, disc widths encompassed a narrower size range of 19–35 cm and were estimated to be primarily young‐of‐year (Clark‐Shen et al., 2025; Wagiyo et al., 2023).

Each ray was placed over a holding tray lined with 1 mm mesh netting. Stomach contents were then collected using the gastric lavage technique described by Myers, Sheaves, and Barnett (2025). All prey items that were successfully flushed from the stomach were removed from the netting using forceps and preserved in 70% ethanol. In cases of rays that were recaptured over multiple trips, gastric lavage was repeated if at least 3 days had passed since the previous capture and the individual appeared in good condition (normal behaviour, no stress colouration), as preliminary work indicated this interval was sufficient for recovery and resumption of normal feeding (Myers, Sheaves, & Barnett, 2025). For stable isotope analysis, a 5 mm diameter biopsy of muscle tissue was collected from the caudal half of the pectoral fin, and tissues were kept on ice. A marker tag was attached to the spiracle of each ray to identify recaptures over repeated site visits. Only *M. toshi* with disc widths >30 cm were large enough for the tag to be attached, so recaptures could only be ascertained from visible scars where tissue samples were previously taken. After a maximum handling time of 15 min, rays were released as close as possible to the site of capture, with brief monitoring to assess recovery. In addition to gastric lavage sampling, three *H. australis* were found deceased on the sand flat on three separate trips, each of which had identifiable prey items in the digestive tract. Stomach and gut contents of these individuals were therefore included for dietary analysis.

### 2.4 Prey availability

Benthic invertebrate communities were sampled three times from September to October 2023. The downstream sand flat was delineated into three habitat strata: subtidal channel edge (3 sites), intertidal sand flat (6 sites) and mangrove edge (3 sites) (Figure 1c). Strata were positioned 25–30 m apart extending eastward from the mangrove edge. Most sampling effort was concentrated on the open sand flat, where rays were frequently observed feeding during rising tides. Benthic invertebrates were targeted across different sediment layers using techniques described by Myers, Sheaves, Abrantes, et al. (2025). Briefly, a sediment corer (20 cm deep × 10 cm diameter) and bait (yabby) pump (60 cm deep × 5 cm diameter) were used to sample infaunal invertebrates when the sand flat was exposed. Five replicate cores were collected per site, and all sediments were pooled for analysis. To sample epibenthic prey, a small beam trawl was dragged across the benthos when the sites were shallowly submerged (rising tides with depth <0.5 m). Trawl effort was quantified by the tow distance (40 m) multiplied by trawl width (0.65 m).

From stomach content analysis (SCA) and benthic survey samples, invertebrate prey organisms were identified visually under a dissecting microscope. Most prey items in the stomachs were wholly intact or could be reliably pieced together into whole organisms. Heavily digested organisms that could not be identified to any level were excluded. Decapod crustaceans were identified to the family level, with some genus and species‐level delineations possible. Amphipods were recorded at the ordinal level. Non‐crustacean organisms including polychaetes, bivalves and gastropods were more common in the benthic surveys but largely absent in the SCA (See Section 3: Section 3.1). Occurrences were therefore retained at Class resolution, as further specifications to family level would not further differentiate the diets of *H. australis* and *M. toshi*.

### 2.5 Statistical analysis

#### 2.5.1 Stomach content analysis

Statistical analyses were run using R version 4.4.1 (R Core Team, 2024). First, cumulative prey curves were constructed for each species to determine whether the number of stomachs were adequate to describe the diet. For the cumulative prey curves and subsequent analyses, classifications to genus and species level were pooled to family level to ensure consistent taxonomic resolution and to facilitate comparisons with other dietary studies (Elston et al., 2020; Myers, Sheaves, Abrantes, et al., 2025; Pardo et al., 2015). Two curves were constructed for each species: (1) all prey categories at family level or higher and (2) frequently consumed prey categories, excluding those with a single occurrence in the dataset. The slope of each line was tested against a slope of zero to determine if a statistical asymptote had been reached (Cortes, 1997; Ebert & Bizzarro, 2007). Significance values for all analyses were interpreted with α = 0.05.

Dietary comparisons were conducted using frequency and count metrics. Frequency of occurrence (%F) was calculated for each species as the number of stomachs containing a particular prey category divided by the total number of stomachs containing prey (Amundsen et al., 1996). Numerical contributions were expressed using the prey‐specific abundance (%Pi) for all prey categories that occurred more than once for a species, calculated as the percentage a given prey category comprises of all prey items for the individuals in which that prey item occurs (Amundsen et al., 1996). When interpreted with %F, these metrics provide further insights into dietary patterns at both the individual and population level by assessing whether dominant (%F > 50) or rare (%F < 50) prey types are consumed following a generalist (%Pi <50) or specialist feeding strategy (%Pi >50) (Amundsen & Sánchez‐Hernández, 2019). Although mass‐ and volume‐based metrics are routinely applied in studies of fish feeding ecology, their use is contingent on both study objectives and limitations regarding robust measurement of prey items (Amundsen & Sánchez‐Hernández, 2019; Baker et al., 2024). Compound indices incorporating bulk components, such as %IRI, were excluded, as these have become recognised as poor indicators of characterising diets (Baker et al., 2014, 2024).

All multivariate analyses were run using the *vegan* package in R (Oksanen et al., 2016). Occurrence data (presence/absence) were used to construct the Jaccard's dissimilarity matrix, as occurrence relies on fewer assumptions than numerical counts and enables more robust comparisons among groups. PERMDISP was performed using the Betadisper function to test whether within‐group dispersion was homogenous for both species, as unequal dispersion can confound differences detected in subsequent analyses (Anderson, 2006). PERMANOVA was then run to compare dietary variability by species, disc width and species * disc width.

To visualise dietary overlap between species, non‐metric multidimensional scaling (NMDS) was applied and overlaid with vectors for each prey category. Prey categories with longer vectors were more strongly associated with the ordination axes, and their directional alignment towards a species ellipse indicated more exclusive patterns of consumption. Significance of each prey category to the ordination axis was assessed via permutation testing (999 permutations) using the ‘envfit’ function. To examine potential size‐related patterns for *H. australis*, two size classes were plotted: <50 cm DW (*n* = 9) and >50 cm DW (*n* = 15). While size ranges of captured rays precluded size‐based comparisons between distinct life stages (e.g., immature vs. mature), limited representation in the 38–55 cm DW range (only one individual) provided clear separation between early and later stage juveniles. For *M. toshi*, all individuals were assessed collectively in the small size class (<50 cm DW).

#### 2.5.2 Stable isotope analysis

For δ^13^C and δ^15^N stable isotope analysis (SIA), muscle samples were stored at −18°C before drying at 60°C for 48 h and homogenised using a mortar and pestle. While urea extraction can be applied in elasmobranch SIA studies, its effects on isotopic values may be inconsistent across species and individuals (Carlisle et al., 2016). Moreover, omitting this step ensured comparability with other studies on juvenile rays (Elston et al., 2020; Myers, Sheaves, Abrantes, et al., 2025; Vaudo & Heithaus, 2011). Lipid extraction was also not conducted in this study, as it is generally not required for rays due to their muscle tissue having a low lipid content (Carlisle et al., 2016; Crook et al., 2019). Samples were analysed at the UC Davis Stable Isotope Facility (USA) with a PDZ Europa ANCA‐GSL elemental analyser interfaced to a PDZ Europa 20–20 isotope ratio mass spectrometer. Results are expressed as per mil (‰) deviations from eight reference standards with a precision of ±0.1‰ (± SD) for both δ^13^C and δ^15^N.

Trophic niche size and overlap were calculated for *H. australis* (*n* = 14) and *M. toshi* (*n* = 15). Samples of *H. australis* were further provisioned into two size classes (<50 and >50 cm DW). With comparisons based on relatively small sample sizes for each group (6 and 8 samples, respectively), we acknowledge these results as preliminary insights into possible ontogenetic variability for this species. Trophic niche breadths were calculated based on the small sample size‐corrected Standard Ellipse Areas (SEAc) using the SIBER package (Jackson et al., 2011). The SEAc overlap (proxy for trophic niche overlap) was then computed as the area of the total overlap between two ellipses divided by the sum of their non‐overlapping areas. To compare SEA sizes between species and size classes (for *H. australis*), the Bayesian SEAs (SEA_B_) were computed, and differences in sizes calculated based on the proportion of times the SEA for one group was larger than that of the other group.

#### 2.5.3 Prey availability

Frequency of occurrence (%F) was calculated for each invertebrate category and sampling method. The percentage numerical contribution of each prey category was calculated by dividing its total abundance by the summed abundance of all prey items and multiplied by 100. To assess variability in invertebrate composition, PERMANOVA models based on presence data were constructed separately for each method (sediment corer, bait pump, trawl) using sampling trip and habitat strata (subtidal edge, open sand flat and mangrove edge) as predictors, followed by post hoc comparisons by stratum. To standardise taxonomic resolution with the SCA, all prey items identified to genus or species were assessed at the family level. Teleosts were commonly caught in the trawl surveys but were excluded from analysis, as they were not expected to be an important prey category for either ray species (Myers, Sheaves, Abrantes, et al., 2025). To contextualise spatial prey variability of key prey taxa for each ray species, densities were calculated for all prey categories with %F > 10 in the diets. Infaunal taxa were quantified from the sediment core samples only by dividing individual counts by sediment volume (individuals m^−3^). For epibenthic taxa, total individuals were divided by the swept surface area of the trawl (individuals m^−2^). Density values were scaled by a factor of 100 for interpretation.

## 3 RESULTS

### 3.1 Stomach content analysis

In total, 24 stomach content samples were collected from *H. australis*, which included 21 from gastric lavage (three from recaptured individuals) and three from deceased specimens. Only four individuals had empty stomachs. A total of 248 individual prey specimens were recovered, and the number of identifiable prey items per stomach ranged from 1 to 35 specimens (mean 10.3 ± 2.0). For *M. toshi*, 22 samples were collected using gastric lavage, and only two individuals had empty stomachs. A total of 110 prey specimens were recovered, and the number of prey items per stomach ranged from 1 to 18 specimens (mean 4.2 ± 0.1). Cumulative prey curves showed that neither species reached a statistical asymptote when all possible prey categories were included (*p* < 0.001 for both) (Figure 2). However, both species reached a statistical asymptote (*H. australis*: *p* = 0.089; *M. toshi*: *p* = 0.092) once categories with single occurrences were excluded. Thus, while more samples would be needed to fully characterise their dietary breadth, sampling effort was considered adequate for generalising the core dietary components of each species.

**FIGURE 2:**
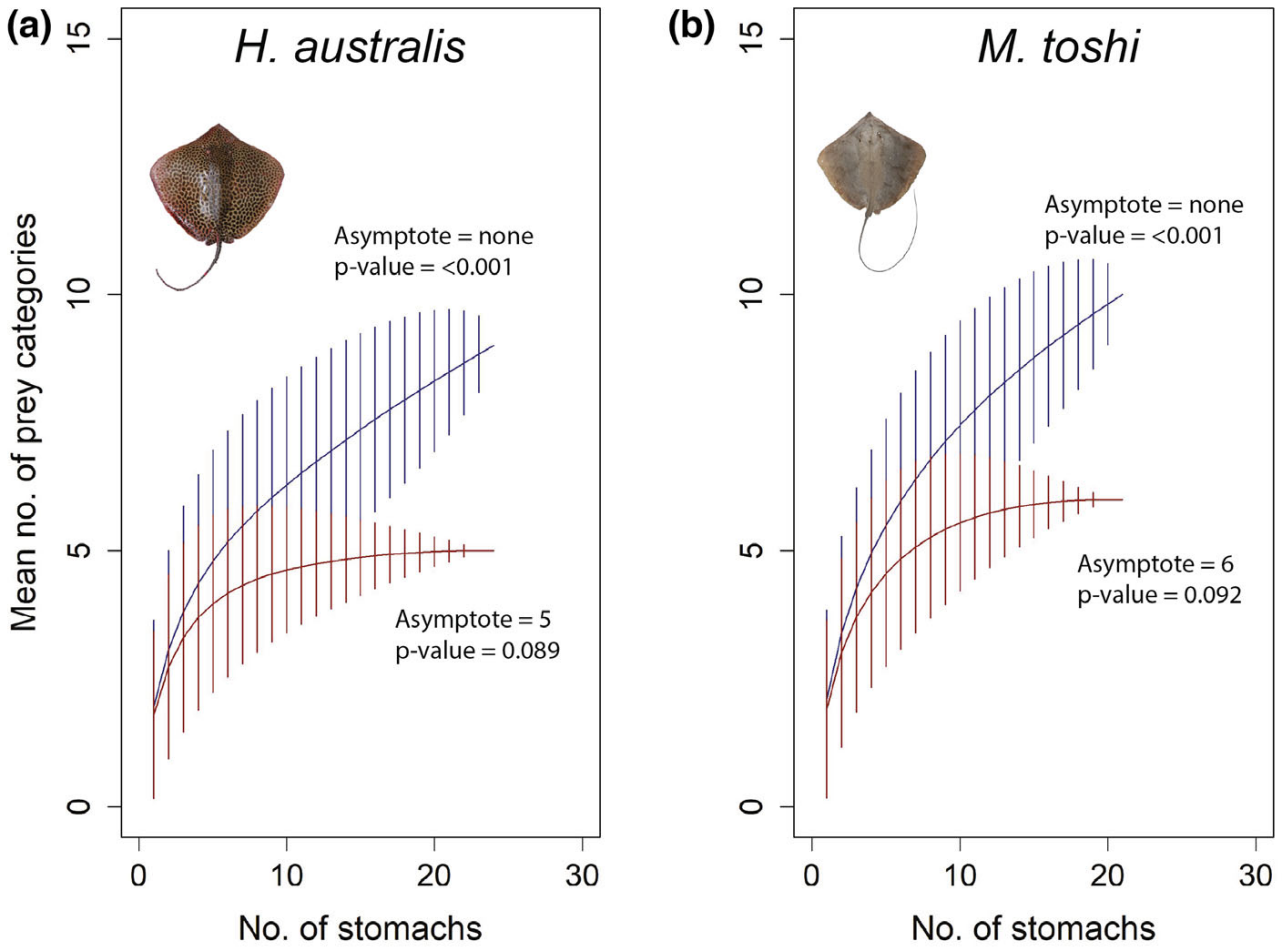
Cumulative prey curves for *Himantura australis* (a) and *Maculabatis toshi* (b). Lines were generated at two resolutions: (1) all identified prey categories in dark blue and (2) excluding categories with only single occurrences in the diet in dark red. Text denotes the asymptote value and significance value when comparing the slope to zero. Ray graphics were adapted from the Australian National Fish Collection.

Diets of *H. australis* were primarily characterised by decapod crustaceans, occurring across all stomachs (Figure 3; Table 1). Brachyuran crabs occurred in 95.8% of samples and primarily consisted of mictyrid crabs (%F = 75.0). High prey‐specific abundance (%Pi = 66.5) indicated specialist consumption by the sampled population. Callianassid shrimp and penaeid prawns each occurred in 37.5% of samples, followed by 20.8% for ocypodid crabs, all with low prey‐specific abundances (%Pi <50). All other prey taxa were present in <10% of ray stomachs. Diets of *M. toshi* were similarly characterised by decapod crustaceans (%F = 90.0), but with greater contributions of epibenthic taxa (Figure 3; Table 1). Caridea, particularly ogyridid shrimp, were dominant (%F = 59.1), with high prey‐specific abundance (%Pi = 65.9) indicative of specialist consumption. Penaeid prawns were also common in the diet (%F = 50.0), followed by portunids (%F = 27.3) and ocypodids (%F = 13.6), with low prey‐specific abundances. Despite amphipods being consumed by few individuals (%F = 13.6), these were consumed in greater abundance (%Pi = 55.6).

**FIGURE 3:**
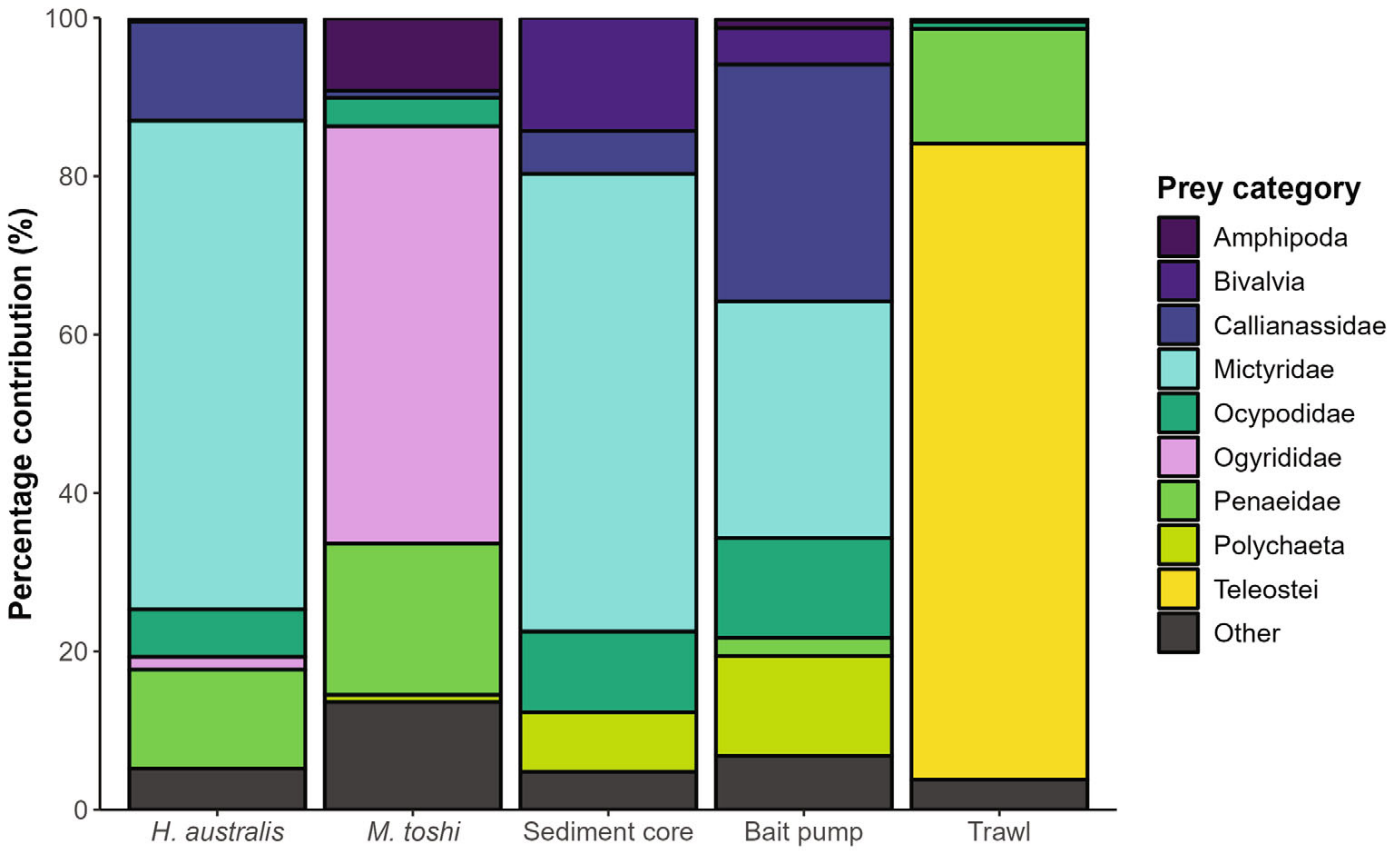
Percentage numerical contributions of prey categories to the diets of *Himantura australis* and *Maculabatis toshi* at Blacksoil Creek, alongside the percentage contributions of invertebrate taxa recorded from sediment core, bait pump and trawl samples. Prey categories constituting >5% of the total numerical contribution across any column are displayed in the legend, while all remaining categories were pooled into “Other”.

**TABLE 1:** Frequency of occurrence (%F) and prey‐specific abundance (%Pi) of prey categories identified in stomach contents of *Himantura australis* and *Maculabatis toshi*.

	*H. australis* (*n* = 24)		*M. toshi* (*n* = 22)	
	%F	%pi	%F	%pi
Prey category				
Decapoda	100.0	88.2	90.9	89.1
Suborder Dendrobranchiata	41.7	38.4	50.0	33.9
Family Sergestidae				
*Acetes* sp.	4.2		0.0	
Family Penaeidae	37.5	25.4	50.0	33.9
*Metapenaeus bennettae*	16.7	18.8	31.8	36.1
*Penaeus merguiensis*	0.0		4.5	
Unknown Penaeid	25.0	35.5	13.6	26.1
Infraorder Axiidea				
Family Callianassidae				
*Trypaea australiensis*	37.5	43.1	4.5	
Infraorder Caridea	4.2		63.6	64.8
Family Alpheidae	0.0		4.5	
Family Ogyrididae				
*Ogyrides* sp.	4.2		59.1	65.9
Unknown shrimp/prawn	0.0		4.5	.
Infraorder Brachyura	95.8	70.8	54.5	18.5
Family Mictyridae				
*Mictyris longicarpus*	75.0	66.5	0.0	
Family Hymenosomatidae	0.0		9.1	8.0
Family Ocypodidae	20.8	20.0	13.6	25.0
*Uca*. sp.	4.1		4.5	
Unknown ocypodid	16.7	13.3	9.1	15.0
Family Macrophthalmidae				
*Macrophthalmus* sp.	8.3	27.3	4.5	
Family Portunidae	4.2		27.3	20.0
*Portunis armatus*	4.2		22.7	20.0
*Scylla serrata*	0.0		4.5	
Unknown crab	8.3	5.7	4.5	
Miscellaneous				
Small crustacea				
Order Amphipoda	4.2		13.6	55.6
Non‐crustacean				
Class Polychaeta	0.0		4.5	

*Note*: The sample size for each species is included in parentheses. Prey‐specific abundance was not calculated for any group with %F of zero or that was present in only one stomach.

Multivariate analysis revealed homogenous dispersion in the SCA (PERMDISP – *F*_1,43_ = 0.73, *p* = 0.394), indicating comparable within‐group variability. Dietary composition differed significantly by species (PERMANOVA – *R* ^2^ = 0.227, *F*_1,41_ = 12.62, *p* = 0.001), although no differences were detected by ray size (*p* = 0.258) or by species × size (*p* = 0.692) (Table S1). Similarly, the NMDS ordination showed minimal overlap between species ellipses due to associations with different prey taxa (Figure 4; Table S2).

**FIGURE 4:**
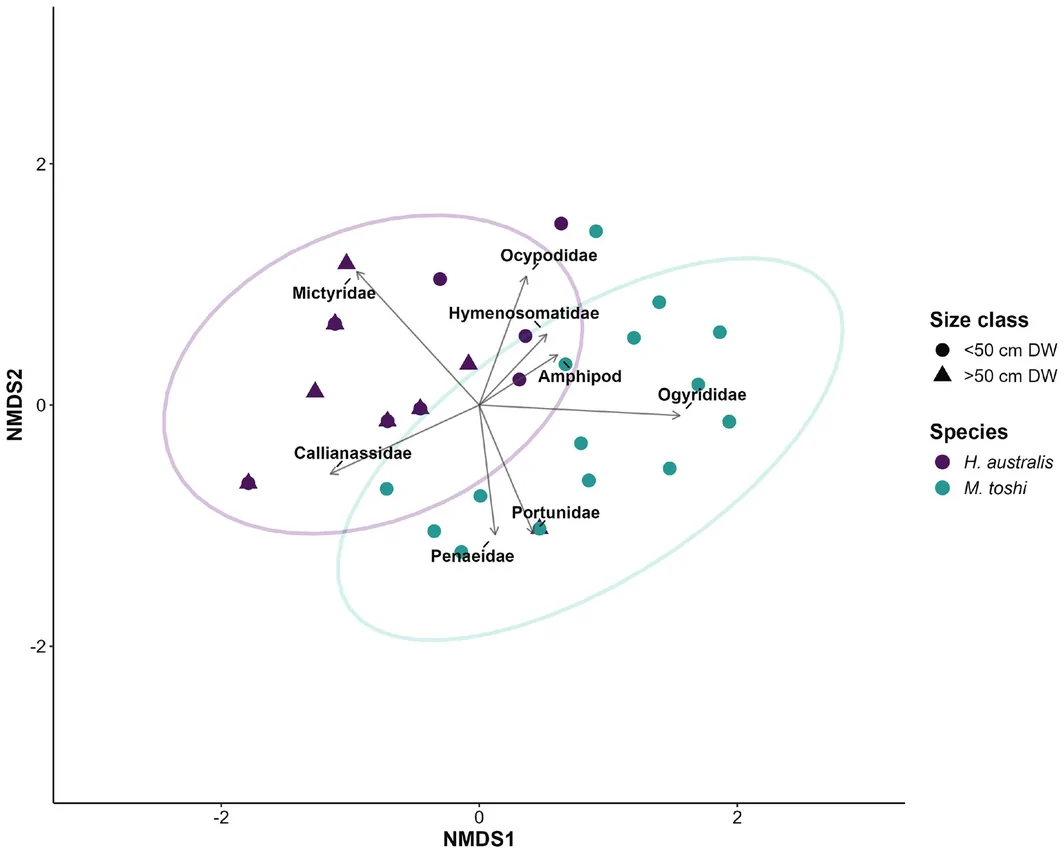
Non‐metric multidimensional scaling (NMDS) ordination plots comparing dietary composition of *Himantura australis* (purple) and *Maculabatis toshi* (turquoise). The 95% confidence interval ellipses are shown for species, with vector associations of various prey categories overlaid within the ordination space. Vectors are drawn for prey categories with a significant correlation with the NMDS axes, and lengths were scaled × 2 to facilitate visualisation. Shapes were used to indicate the two size classes for *H. australis*, which shared high overlap within the space.

### 3.2 Stable isotope analysis

Mean values of δ^13^C ranged from −16.7 to −14.5‰ and mean δ^15^N from 8.4 to 9.6‰ across all samples (Table 2). Significant negative relationships were observed between δ^13^C values and disc width for both *H. australis* (*R* ^2^ = 0.296, *p* = 0.0444) and *M. toshi* (*R* ^2^ = 0.277, *p* = 0.0436) (Figure 5a,b). For δ^15^N, values showed no significant relationship with disc width for either species (*H. australis: p* = 0.0662; *M. toshi: p* = 0.7857), indicating no change in trophic position over the size ranges tested (Figure 5c,d).

**TABLE 2:** Details of rays analysed for stable isotope analysis including disc size class, mean δ^13^C and δ^15^N (± SD), C:N ratios and trophic niche sizes.

Species	Size	*N*	δ^13^C (‰)	δ^15^N (‰)	C:N	SEA (‰^2^)	SEAc (‰^2^)
*Himantura australis*	All	14	−15.7 ± 1.4	8.8 ± 1.1	3.2 ± 0.2	4.6 (4.6–4.6)	5
	<50 cm	6	−14.5 ± 0.9	9.3 ± 1.4	3.3 ± 0.1	4.2 (−2.4–34.8)	5.2
	>50 cm	8	−16.7 ± 0.9	8.4 ± 0.5	3.2 ± 0.2	1.0 (−0.2–6.3)	1.1
*Maculabatis toshi*	<50 cm	15	−15.2 ± 0.7	9.6 ± 0.2	3.3 ± 0.1	0.4 (−0.2–1.8)	0.5

*Note*: For SEA_B_ (Bayesian standard ellipse area), means of SEA are reported with the 95% credible intervals (in parentheses), while SEAc denotes the small size‐corrected standard ellipse area (‰^2^).

**FIGURE 5:**
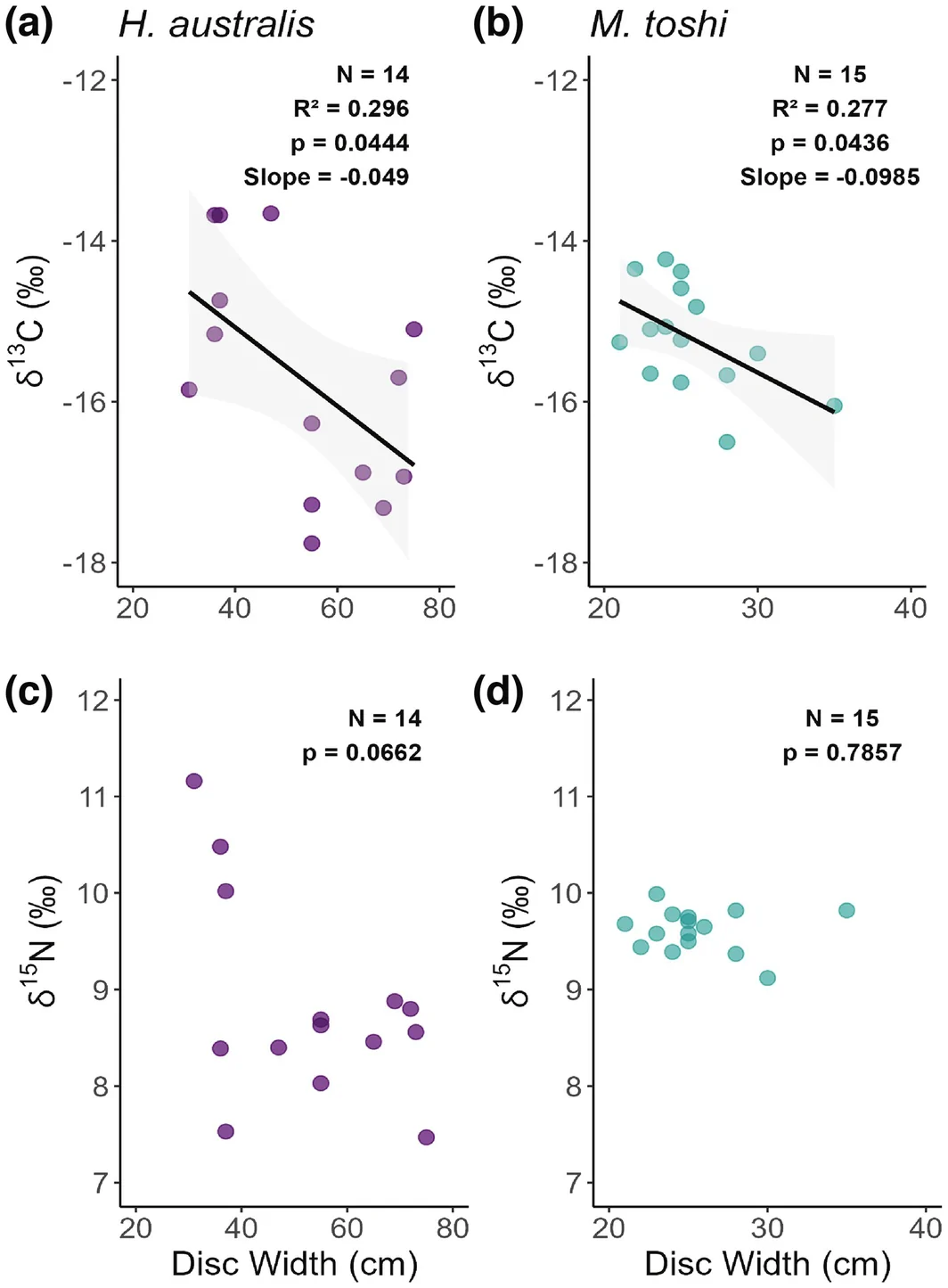
Linear regression models examining relationships between disc width and δ^13^C (a,b) and δ^15^N (c,d) for *Himantura australis* and *Maculabatis toshi* analysed from Blacksoil Creek. Sample sizes and *p*‐values from the regression models are displayed for all panels, and *R* ^2^ and slopes are displayed for each significant relationship.

*Himantura australis* exhibited a larger trophic niche size than *M. toshi* (Figure 6a), and species shared only 9.5% overlap in SEAc niche areas (Figure 6b). Subdividing *H. australis* into two size classes showed that *M. toshi* and larger *H. australis* >50 cm DW both had narrower trophic niches than *H. australis* <50 cm DW (Figure 6c). Larger *H. australis* were the most differentiated group, showing no trophic overlap with *M. toshi* or the smaller size class. Only 6.9% niche overlap occurred between smaller *H. australis* and *M. toshi* (Figure 6d).

**FIGURE 6:**
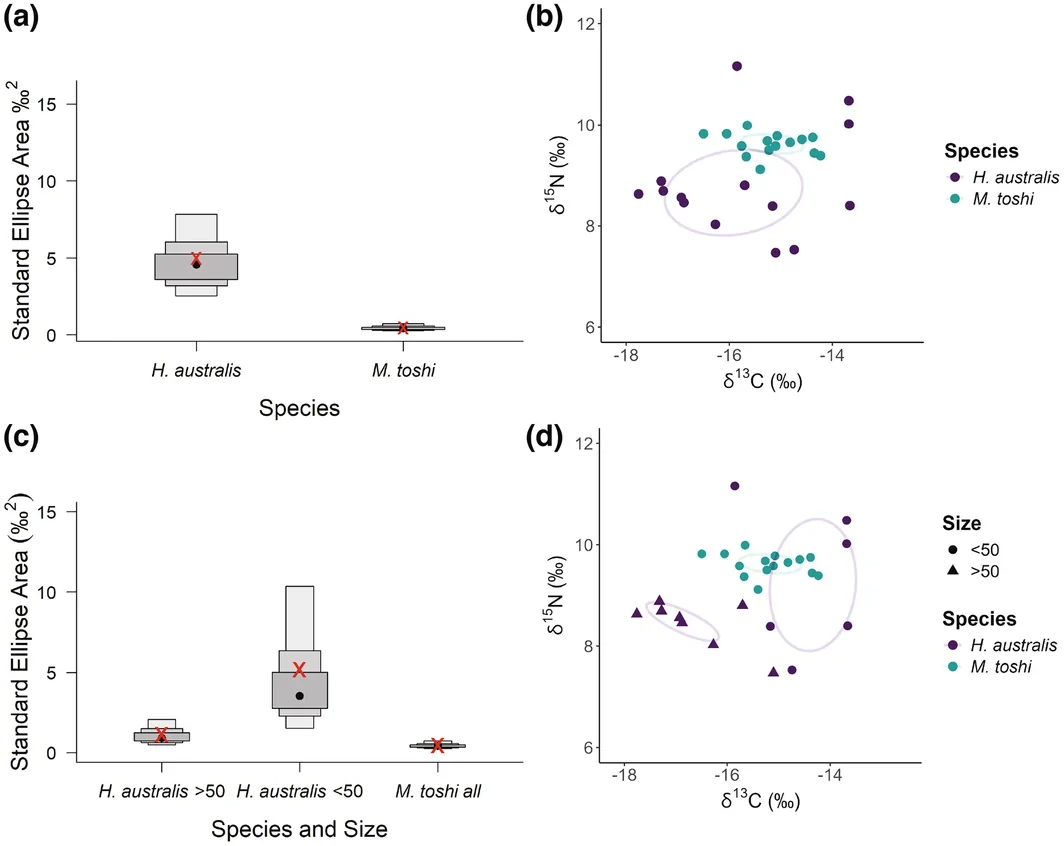
Trophic niche size and overlap between *Himantura australis* and *Maculabatis toshi*. Density plots illustrate the standard ellipse area (SEA) of each species, with a red ‘x’ denoting the small‐size‐corrected SEA (SEA_c_) value (a). Isotopic biplots display δ^13^C and δ^15^N values with the 40% standard ellipse areas for each species (b). These figures are also shown between *M. toshi* and the two size classes of *H. australis* (c,d).

### 3.3 Prey availability

In total, 476 benthic fauna specimens were collected across the study area, which varied among habitat strata (Table 3). Ten families of decapod crustaceans were recorded, of which seven were present in the SCA. Other invertebrates, including polychaetes, bivalves and gastropods, were also present, as well as teleosts. In sediment core samples, 57.8% of sampled individuals were mictyrid crabs, with lesser contributions of bivalves, callianassids, ocypodids and polychaetes (Figure 3). Bait pump samples contained more balanced contributions of mictyrid crabs and callianassids (29.9% for both), but taxonomic representation was similar to the sediment cores. Trawl samples were highly dominated by teleosts (80.3% of total), along with minor contributions of penaeid prawns (14.5%) (Figure 3).

**TABLE 3:** Frequency of occurrence (%F) of prey items identified in the benthic fauna sampling from September to October 2023.

Prey category	Intertidal sand flat	Mangrove mud bank	Subtidal channel
Decapoda	72.2	70.4	33.3
Suborder Dendrobranchiata	44.4	22.2	18.5
Family Sergestidae			
*Acetes sibogae*	1.9	0.0	3.7
Family Penaeidae	9.3	18.5	18.5
*Metapenaeus bennettae*	0.0	9.3	5.6
*Penaeus merguiensis*	7.4	18.5	11.1
Infraorder Axiidea			
Family Callianassidae			
*Trypaea australiensis*	33.3	3.7	0.0
Infraorder Caridea			
Family Alpheidae	1.9	0.0	0.0
Infraorder Anomura			
Family Diogenidae	0.0	3.7	14.8
Infraorder Brachyura	53.7	51.9	22.2
Family Grapsidae	1.9	3.7	0.0
Family Hymenosomatidae	0.0	0.0	3.7
Family Mictyridae			
*Mictyris longicarpus*	42.6	33.3	0.0
Family Ocypodidae	16.7	37.0	0.0
Family Portunidae			
*Portunis armatus*	1.9	0.0	3.7
Miscellaneous			
Class Polychaeta	11.1	11.1	29.6
Small crustacea			
Order Amphipoda	1.9	0.0	3.7
Class Bivalvia	16.7	0.0	22.2
Class Gastropoda	7.4	0.0	0.0
Benthic fishes (Teleostei)	25.9	25.9	29.6

*Note*: Results are listed for each habitat stratum and pooled across sampling methods (sediment core, bait pump, trawl).

Benthic invertebrate communities varied among habitat strata for all three sampling methods (PERMANOVA – *p* < 0.01 for all), while sampling trip was only marginally significant among trawl samples (*p* = 0.049) (Table S3). Samples that were collected using sediment cores differed between the subtidal edge vs. intertidal sand flat (*p* = 0.009), subtidal edge vs. mangrove edge (*p* = 0.003), and intertidal sand flat vs. mangrove edge (*p* = 0.012) (Table S4). For the bait pump, invertebrate composition varied only between the subtidal edge vs. intertidal sand flat (*p* = 0.006). Despite significance in the PERMANOVA, post hoc testing revealed no differences between any stratum pairs among trawl samples.

In the sediment core samples, mictyrid crabs had high densities along the mangrove edge (52.4 ± 44.8 individuals 100 m^−3^) and intertidal sand flat (34.0 ± 36.0 individuals 100 m^−3^), but were absent from the subtidal channel (Table 4). Callianassids only occurred on the intertidal sand flat (5.7 ± 10.0 individuals 100 m^−3^). Ocypodid crabs were most abundant along the mangrove edge (14.2 ± 16.2 individuals 100 m^−3^) and were absent from the subtidal edge. Densities of epibenthic fauna were relatively low in the trawl samples, with penaeid prawns most abundant on the mangrove edge (3.4 ± 7.1 individuals 100 m^−2^) and amphipods along the subtidal edge (0.4 ± 1.3 individuals 100 m^−2^). Portunid crabs were only encountered on the intertidal sand flat (0.4 ± 1.2 individuals 100 m^−2^) (Table 4).

**TABLE 4:** Mean densities (± SD) of key prey taxa (those with frequency of occurrence >10% from stomach content analysis) are compared across three habitat strata on the downstream intertidal flat.

Method	Type	Prey category	Intertidal sand flat	Mangrove edge	Subtidal channel edge
Core	Infauna	Mictyridae	34.0 ± 36.0	52.4 ± 44.8	0
Core	Infauna	Ocypodidae	3.5 ± 8.5	14.2 ± 16.2	0
Core	Infauna	Callianassidae	5.7 ± 10.0	0	0
Trawl	Epibenthic	Amphipoda	0	0	0.4 ± 1.3
Trawl	Epibenthic	Penaeidae	0.9 ± 2.1	3.4 ± 7.1	0.4 ± 1.3
Trawl	Epibenthic	Portunidae	0.4 ± 1.2	0	0

*Note*: Sediment core samples are given as individuals 100 m^−3^ and trawl samples as individuals 100 m^−2^. All prey taxa are given at the family level, with the exception of Order Amphipoda.

## 4 DISCUSSION

Within the Blacksoil Creek estuary inlet, diets of *H. australis* and *M. toshi* were both dominated by decapod crustaceans. However, differences in dietary composition and trophic niches supported interspecific resource partitioning between these co‐occurring dasyatid species. Notably, non‐crustacean taxa including polychaetes, molluscs and teleosts were present across the study area but were largely absent from the diets of either species, indicating they are crustacean specialists rather than generalist consumers of all available resources.

General consumption of decapod crustaceans including prawns and brachyuran crabs has been previously documented for *H. australis* from nearshore environments in northern Australia (Myers, Sheaves, Abrantes, et al., 2025; O'Shea, Thums, et al., 2013; Vaudo & Heithaus, 2011). In this study, individuals frequently consumed deep‐burrowing crustacean taxa, particularly callianassid ghost shrimp *Trypaea australiensis* and soldier crabs *Mictyris longicarpus*. Where present in the stomach contents, both could occur in high quantities. For example, 35 soldier crabs were flushed from the stomach of one *H. australis* in the <50 cm DW size class. Another sample from a larger individual (80 cm DW) contained nine whole ghost shrimp, with few other prey items present. Both prey taxa are commonly found in high but spatially variable densities on intertidal sand and mud flats (Dittmann, 2000; Tamaki & Ingole, 1993) and were among the most abundant prey sampled at Blacksoil Creek. Their patchy but locally dense distributions suggest that *H. australis* may opportunistically exploit highly concentrated density patches while feeding in sand and mud flat habitats, consistent with a generalist feeding strategy.

Previous benthic surveys at nearby Lucinda Beach showed that ghost shrimp and soldier crabs were patchily distributed in sediment cores across the intertidal zone and less abundant than other invertebrate groups such as bivalves, gastropods, polychaetes and ocypodid crabs (Myers, Sheaves, Abrantes, et al., 2025). These taxa contributed little to diets of *H. australis* at Lucinda Beach, occurring in only 12% and 4% of stomachs, respectively. Differences between locations may therefore reflect differences in invertebrate prey communities. Alternatively, it is possible that ontogenetic resource partitioning may have played a partial role, given that individuals sampled at Lucinda Beach were overall smaller (mean 37.9 ± 1.3 cm DW) than rays at Blacksoil Creek (mean 52.7 ± 3.2 cm DW) (Myers, Sheaves, Abrantes, et al., 2025).

Burrowing crustaceans were largely absent from the diet of *M. toshi*, which contributed to low trophic overlap with *H. australis*. Rather, these individuals showed greater consumption of epibenthic crustacean taxa, particularly Caridean shrimp and penaeid prawns. Prevalence of penaeid prawns has been similarly documented in other studies on young‐of‐year size classes (Myers, Sheaves, Abrantes, et al., 2025) and larger individuals (Vaudo & Heithaus, 2011), indicating they are a consistent resource across nearshore nursery contexts. SIA from these prior studies similarly documented smaller isotopic niches for *M. toshi* than *H. australis*, supporting a more specialised diet. Lower densities of epibenthic invertebrates in the trawl surveys likely reflect their greater mobility and capacity to distribute across the benthos during tidal fluxes (Hill et al., 1982; Silva et al., 2014). Consequently, while *H. australis* may benefit from high‐density patches of infaunal prey, sparser distributions of epibenthic taxa could mean that *M. toshi* encounters prey more opportunistically. Despite being common in the diets of *M. toshi*, *Ogyrides* shrimp were absent from all invertebrate sampling methods. While this result could indicate high specialisation of a potentially uncommon resource, it is uncertain whether this prey type inhabits other microhabitats which were not represented here, or were otherwise excluded by our methods.

Ontogenetic dietary shifts in elasmobranch populations are common as juveniles gain access to new habitats and develop new foraging capabilities (Barbini & Lucifora, 2012; Grubbs, 2010; Lowry & Motta, 2008), which either causes the diet to expand to include more prey types as body size increases or narrow to specialise on fewer profitable prey types (Barbini et al., 2018; McInturf et al., 2025; Priester et al., 2024). Ontogenetic changes are often reflected in stable isotope values, where shifts in δ^13^C show changes in foraging habitat or basal carbon sources, whilst changes in δ^15^N reflect transitions to higher or lower trophic positions (Hussey et al., 2012). Both ray species showed significant negative relationships between δ^13^C and disc width but no relationship with δ^15^N, indicating potential shifts in diet/microhabitat without altering trophic position. Partitioning *H. australis* into small and large size classes provided greater insights into size‐related effects on their trophic ecology. Whereas marginal niche overlap was present with *M. toshi* when samples were assessed collectively, this was only the case for the smaller size class *H. australis* (<50 cm DW). In contrast, larger *H. australis* (>50 cm DW) displayed a significantly narrower niche breadth than smaller conspecifics, indicating that intra‐ and interspecific overlap may diminish as *H. australis* age and occupy new dietary niche spaces within the estuary. With such narrow size variability for *M. toshi*, the inclusion of larger individuals would be needed to verify if such trophic shifts occur beyond early juvenile stages.

Prey selectivity in elasmobranchs can stem from various morphological and behavioural factors that subsequently influence foraging capabilities (Dean et al., 2007; Kolmann et al., 2018; Motta et al., 2008; Wilga et al., 2007). For suction feeding rays, gape size and suction power scale positively with body size, enabling larger individuals to generate greater negative pressures in the buccal cavity and displace more sediment with their rostra and pectoral fins (Kolmann et al., 2015; Lowry & Motta, 2008). While both species appear to share similar morphologies, the larger body sizes of *H. australis* may give them greater excavation capabilities than *M. toshi* and smaller conspecifics. Such linkages can be better understood when considering the distributions of their target prey in the sediment column. Soldier crabs, which were prevalent in the stomachs of *H. australis*, generally burrow up to ~15 cm beneath the sediment surface (Rossi & Chapman, 2003), whereas callianassid ghost shrimp can construct burrows exceeding 50 cm deep (Kirby & Wong, 2025), which supports their greater representation in the bait pump cores. Despite their high abundances across the intertidal zone, these prey taxa may therefore be more accessible to larger individuals capable of overturning larger volumes of sediment.

Aerial drone videos from Blacksoil Creek recorded in 2020–2022 showed high abundances of *H. australis* exhibiting excavation‐style foraging, where rays were routinely observed displacing large amounts of sediment along shallow sand flat edges, particularly in areas containing high burrow densities (Videos S1 and S2). Rays continued to forage over the newly submerged sand flat areas closer to the mangroves as these became available during rising tides. Excavations were similarly observed for this species at Lucinda Beach (Crook et al., 2022), indicating that rays use this strategy consistently when foraging over sandy‐bottom substrates. In contrast, smaller body sizes of *M. toshi* may necessitate the use of less disruptive surface feeding, creating greater dependencies on epibenthic prey (Myers, Sheaves, Abrantes, et al., 2025); however, more research is still needed to characterise their foraging behaviours.

## 5 CONCLUSIONS

With estuaries and associated species facing intensifying pressures from anthropogenic activities (Dhanjal‐Adams et al., 2016; Sheaves et al., 2014), the ongoing risk of habitat disruption and decline necessitates closer evaluations of their habitat value and functional uses. Moreover, trophic ecology assessments from these systems contribute to our understanding of biotic drivers that influence habitat use and broader community dynamics. Our findings support the value of estuary inlets as important feeding grounds for juvenile rays (Constance et al., 2024) while highlighting several possible directions of future research. Interpreting stomach contents alongside stable isotope analysis provided complementary evidence of dietary resource partitioning and trophic interactions among related ray species. Incorporating prey availability data is another critical component for contextualising dietary variability across different spatial contexts (Barnett et al., 2010; O'Shea, Thums, et al., 2013; Pardo et al., 2015), yet is often neglected and/or difficult in elasmobranch trophic studies. Its inclusion here suggests that while both species target locally abundant crustacean taxa, resources may be partitioned across different sediment layers, requiring further consideration for how diets are influenced by both prey availability and accessibility.

This study provides preliminary insights into the dietary habits of each species in tropical estuaries, although knowledge gaps still surround the resource requirements of *H. australis* and *M. toshi* over ontogeny. Small sample sizes for the SCA warrant further validation to fully characterise their diets and to account for factors such as seasonal and spatial variations in prey availability. Likewise, SIA supported ontogenetic trophic shifts for *H. australis*, but further investigations are still needed to understand at which sizes key dietary transitions occur. The absence of comparable size classes of *M. toshi* precluded similar inspection for this species. Consequently, we could not determine whether interspecific variability reflected fundamental species‐level differences or was confounded by ray size, given that the site is inhabited by largely non‐overlapping size classes. Thus, it remains unclear whether the dietary requirements of *M. toshi* would change significantly over ontogeny or how this could affect trophic interactions with *H. australis*, where larger size classes co‐occur. Additional information on fine‐scale habitat use would be needed to confirm that rays are primarily foraging within the surveyed area or supplement resources from outside the inlet. Moreover, the absence of adult rays at Blacksoil Creek raises questions about whether older juveniles leave estuaries for deeper benthic habitats and which factors trigger their departure.

Lastly, linking differences in foraging strategies among ray species with functional roles and impacts remains a key gap in batoid research, with the integration of behavioural data largely absent beyond a few localised studies (Crook et al., 2022; Myers et al., 2026). Our results from Blacksoil Creek suggest that bioturbation, and any subsequent effects on sand flat geomorphology (Nauta et al., 2024), are performed more exclusively by *H. australis* rather than being shared equally with other species. Overall, continued investigations into trophic ecology, prey distributions and foraging behaviour at the species level will be valuable for understanding resource use and requirements, as well as linking rays to broader estuarine processes.

## AUTHOR CONTRIBUTIONS

**Jaelen Myers**: Conceptualisation; data collection; data analysis; manuscript preparation; funding. **Marcus Sheaves**: Conceptualisation; manuscript editing; project supervision. **Adam Barnett**: Conceptualisation; manuscript editing; project supervision.

## FUNDING INFORMATION

This research was funded by the Holsworth Wildlife Research Endowment and the Ecological Society of Australia.

## Supporting information


**Video S1.** One *Himantura australis* using excavation foraging over fine sand.

**Video S2.** Numerous *Himantura australis* foraging along the intertidal flat edges during an early rising tide.

**Table S1.** Results of PERMANOVA testing for variability in the dietary composition of *Himantura australis* and *Maculabatis toshi*.
**Table S2.** Correlations of 12 prey categories with the NMDS ordination. NMDS1 and NMDS2 give the vector coordinates for each prey category; vector length was calculated using the Euclidean distance, which was scaled by a factor of 2 for graphical display. *R* ^2^ indicates the proportion of variance in the ordination explained by each prey vector, and bolded *p*‐values indicate prey categories with a significant association with the ordination axes (permutation test, 999 permutations, *p* < 0.05).
**Table S3.** PERMANOVA table showing the effects of habitat stratum and sampling trip on benthic invertebrate composition (using presence data). Bolded *p*‐values denote statistical significance (*p* < 0.05).
**Table S4.** Results of post hoc analysis comparing differences in benthic invertebrate communities between three habitat strata at Blacksoil Creek, sampled using three methods. *p*‐values were adjusted for multiple comparisons using the Bonferroni correction, and significant values are denoted in bold text.

## Data Availability

The data that support the findings of this study are available from the corresponding author upon reasonable request.
