# Supplementary material for: Insights into diet and niche separation in the context of prey availability between two sympatric whiprays in a tropical intertidal estuary
Source: J Fish Biol. 2026 Aug 2;109(4):2248–62. doi: 10.1111/jfb.70596 (PMC13675496; doi:10.1111/jfb.70596)
Supplement: Supplementary file 1 [file JFB-109-2248-s001.docx]

**Supplementary Materials**

**Insights into diet and niche separation in the context of prey availability between two sympatric whiprays in a tropical intertidal estuary**

Jaelen Myers, Marcus Sheaves, Adam Barnett

Table S1. Results of PERMANOVA testing for variability in the dietary composition of *Himantura australis* and *Maculabatis toshi*.

| **Factor** | **DF** | **SS** | **R^2^** | **F-statistic** | **p-value** |
| --- | --- | --- | --- | --- | --- |
| Species | 1 | 3.55 | 0.23 | 12.621 | **0.001** |
| Disc width | 1 | 0.36 | 0.02 | 1.269 | 0.258 |
| Species:Disc width | 1 | 0.19 | 0.01 | 0.679 | 0.692 |
| Residual | 41 | 11.53 | 0.74 | NA | NA |
| Total | 44 | 15.62 | 1 | NA | NA |

Table S2. Correlations of twelve prey categories with the NMDS ordination. NMDS1 and NMDS2 give the vector coordinates for each prey category; vector length was calculated using the Euclidean distance, which was scaled by a factor of 2 for graphical display. R² indicates the proportion of variance in the ordination explained by each prey vector, and bolded p-values indicate prey categories with a significant association with the ordination axes (permutation test, 999 permutations, p < 0.05).

| **Prey category** | **NMDS1** | **NMDS2** | **Vector length** | **Vector length (* 2)** | **R^2^** | **p-value** |
| --- | --- | --- | --- | --- | --- | --- |
| Ogyrididae | 1.554009 | -0.08671 | 1.56 | 3.11 | 0.61 | **0.001** |
| Mictyridae | -0.94953 | 1.105303 | 1.46 | 2.91 | 0.53 | **0.001** |
| Callianassidae | -1.15334 | -0.56872 | 1.29 | 2.57 | 0.41 | **0.001** |
| Portunidae | 0.419719 | -1.06543 | 1.15 | 2.29 | 0.33 | **0.001** |
| Ocypodidae | 0.365227 | 1.065247 | 1.13 | 2.25 | 0.32 | **0.001** |
| Penaeidae | 0.123612 | -1.07483 | 1.08 | 2.16 | 0.29 | **0.003** |
| Hymenosomatidae | 0.522646 | 0.586664 | 0.79 | 1.57 | 0.15 | **0.01** |
| Amphipod | 0.605922 | 0.415799 | 0.73 | 1.47 | 0.14 | **0.049** |
| Macrophthalmidae | 0.179183 | 0.654585 | 0.68 | 1.36 | 0.12 | 0.086 |
| Sergestidae | -0.19251 | 0.55126 | 0.58 | 1.17 | 0.09 | 0.071 |
| Alpheidae | -0.02394 | -0.5325 | 0.53 | 1.07 | 0.07 | 0.265 |
| Polychaete | -0.06131 | -0.46267 | 0.47 | 0.93 | 0.05 | 0.305 |

Table S3. PERMANOVA table showing the effects of habitat stratum and sampling trip on benthic invertebrate composition (using presence data). Bolded p-values denote statistical significance (p<0.05).

| **Method** | **Factor** | **DF** | **SS** | **R^2^** | **F-statistic** | **p-value** |
| --- | --- | --- | --- | --- | --- | --- |
| Core | Stratum | 2 | 2.98 | 0.29 | 6.44 | **0.001** |
|  | Trip | 2 | 0.74 | 0.07 | 1.60 | 0.117 |
|  | Residual | 29 | 6.70 | 0.64 | NA | NA |
|  | Total | 33 | 10.43 | 1.00 | NA | NA |
|  |  |  |  |  |  |  |
| Bait pump | Stratum | 2 | 2.51 | 0.25 | 4.33 | **0.002** |
|  | Trip | 2 | 0.61 | 0.06 | 1.06 | 0.367 |
|  | Residual | 24 | 6.97 | 0.69 | NA | NA |
|  | Total | 28 | 10.16 | 1.00 | NA | NA |
|  |  |  |  |  |  |  |
| Trawl | Stratum | 2 | 0.87 | 0.22 | 2.21 | **0.039** |
|  | Trip | 2 | 0.87 | 0.22 | 2.23 | **0.049** |
|  | Residual | 12 | 2.35 | 0.59 | NA | NA |
|  | Total | 16 | 3.97 | 1.00 | NA | NA |

Table S4. Results of post-hoc analysis comparing differences in benthic invertebrate communities between three habitat strata at Blacksoil Creek, sampled using three methods. P-values were adjusted for multiple comparisons using the Bonferroni correction, and significant values are denoted in bold text.

| Method | Comparison | R^2^ | F-statistic | p-value (adj.) |
| --- | --- | --- | --- | --- |
| core | subtidal vs. intertidal | 0.21 | 6.144 | **0.009** |
|  | subtidal vs. mangrove | 0.40 | 9.899 | **0.003** |
|  | intertidal vs. mangrove | 0.14 | 3.952 | **0.012** |
|  |  |  |  |  |
| bait pump | subtidal vs. intertidal | 0.24 | 6.225 | **0.006** |
|  | subtidal vs. mangrove | 0.25 | 3.388 | 0.072 |
|  | mangrove vs. intertidal | 0.13 | 3.354 | 0.063 |
|  |  |  |  |  |
| trawl | subtidal vs. mangrove | 0.24 | 2.526 | 0.147 |
|  | subtidal vs. intertidal | 0.10 | 1.079 | 1.000 |
|  | mangrove vs. intertidal | 0.15 | 1.799 | 0.969 |
